# Effective Removal of Pb(II) Ions by Electrospun PAN/Sago Lignin-Based Activated Carbon Nanofibers

**DOI:** 10.3390/molecules25133081

**Published:** 2020-07-06

**Authors:** Nurul Aida Nordin, Norizah Abdul Rahman, Abdul Halim Abdullah

**Affiliations:** 1 Department of Chemistry, Faculty of Science, Universiti Putra Malaysia, Serdang 43400, Selangor, Malaysia; aida_nurul93@yahoo.com (N.A.N.); halim@upm.edu.my (A.H.A.); 2 Materials Processing and Technology Laboratory, Institute of Advanced Technology, Universiti Putra Malaysia, Serdang 43400, Selangor, Malaysia; 3 Materials Synthesis and Characterization Laboratory, Institute of Advanced Technology, Universiti Putra Malaysia, UPM, Serdang 43400, Selangor, Malaysia

**Keywords:** carbon nanofibers, activated carbon nanofibers, sago lignin, PAN, adsorption, heavy metal removal, adsorbent, Pb(II) ions

## Abstract

Heavy metal pollution, such as lead, can cause contamination of water resources and harm human life. Many techniques have been explored and utilized to overcome this problem, with adsorption technology being the most common strategies for water treatment. In this study, carbon nanofibers, polyacrylonitrile (PAN)/sago lignin (SL) carbon nanofibers (PAN/SL CNF) and PAN/SL activated carbon nanofibers (PAN/SL ACNF), with a diameter approximately 300 nm, were produced by electrospinning blends of polyacrylonitrile and sago lignin followed by thermal and acid treatments and used as adsorbents for the removal of Pb(II) ions from aqueous solutions. The incorporation of biodegradable and renewable SL in PAN/SL blends fibers produces the CNF with a smaller diameter than PAN only but preserves the structure of CNF. The adsorption of Pb(II) ions on PAN/SL ACNF was three times higher than that of PAN/SL CNF. The enhanced removal was due to the nitric acid treatment that resulted in the formation of surface oxygenated functional groups that promoted the Pb(II) ions adsorption. The best-suited adsorption conditions that gave the highest percentage removal of 67%, with an adsorption capacity of 524 mg/g, were 40 mg of adsorbent dosage, 125 ppm of Pb(II) solution, pH 5, and a contact time of 240 min. The adsorption data fitted the Langmuir isotherm and the pseudo-second-order kinetic models, indicating that the adsorption is a monolayer, and is governed by the availability of the adsorption sites. With the adsorption capacity of 588 mg/g, determined via the Langmuir isotherm model, the study demonstrated the potential of PAN/SL ACNFs as the adsorbent for the removal of Pb(II) ions from aqueous solution.

## 1. Introduction

Heavy metals can contribute to environmental problems, which could have an impact on humans due to its toxicity. The heavy metal pollution could bring an implication to the aquatic life bodies, natural water bodies, and possibly get trapped in the soil through bioaccumulation [1]. Its high solubility in the aquatic environment allows it to be absorbed by living organisms, and large concentrations of heavy metals might accumulate in the human body once they enter our food chain and caused severe health disorders [2]. Lead has been utilized as industrial raw materials for battery manufacturing, printing, pigments, fuels, photographic documents, and dyeing [3]. Lead is a metal which is considered the highest of the environmental concerns [4,5]. A few methods have been developed for the removal of lead from wastewater such as electrochemical treatment, ion exchange, coagulation, reverse osmosis, membrane filtration, adsorption, etc. [6]. Previous studies have shown water treatment by adsorption to be the most convenient, simple, economical, and effective to be used in practical applications [7,8]. Numerous adsorbents have been developed for the treatment of wastewater such as kaolinite clay [9,10], zeolites [11], agricultural waste [12], polymeric materials [13,14] and carbon-based materials [15,16,17,18]. Among these adsorbents, carbon-based materials were found to be effective adsorbents for the removal of heavy metal due to their well-developed internal pore structures, large specific surface area, and presence of surface functional groups [18]. 

Natural polymers received massive attention from the researchers in order to reduce the consumption of petroleum-based polymers [19]. Cellulose, starch, and lignin are among the natural polymers which possess exciting characteristics such as biodegradable, non-toxic, and low cost. In Malaysia, 52,000 tons of sago starch was produced in 2011, a 64% increase over 2001 [20]. The sago starch extraction produces large quantities of residue for which no effective disposal method has been developed [21]. The waste generated from the process of sago starch mainly consists of cellulose and lignin [22]. Besides, lignin has the potential in the production of carbon-based materials due to its structure which consists of many aromatic components.

Synthesis of carbon materials particularly CNFs from renewable resources such as biomass has scarcely been reported. Mamun, Ahmed [23] implemented the impregnation method to produce CNFs by utilizing oil palm shell as the sources of carbon. However, there is no reported literature on producing CNFs derived from the use of sago palm biomass. To the best of our knowledge, there is no report on the production of CNFs by using electrospinning for the production of CNFs from palm waste or sago waste. In this study, an adsorbent was prepared from the electrospun fibers of blends of PAN and sago lignin isolated from sago waste. Incorporation of sago lignin (SL) in the fibers reduced the usage of petroleum-based polyacrylonitrile (PAN). After thermal and acid treatments, the prepared ACNFs was used as an adsorbent for the removal of Pb(II) ions from aqueous solution. 

## 2. Results and Discussion

### 2.1. Optimization of Electrospun PAN Nanofibers

**Figure 1:**
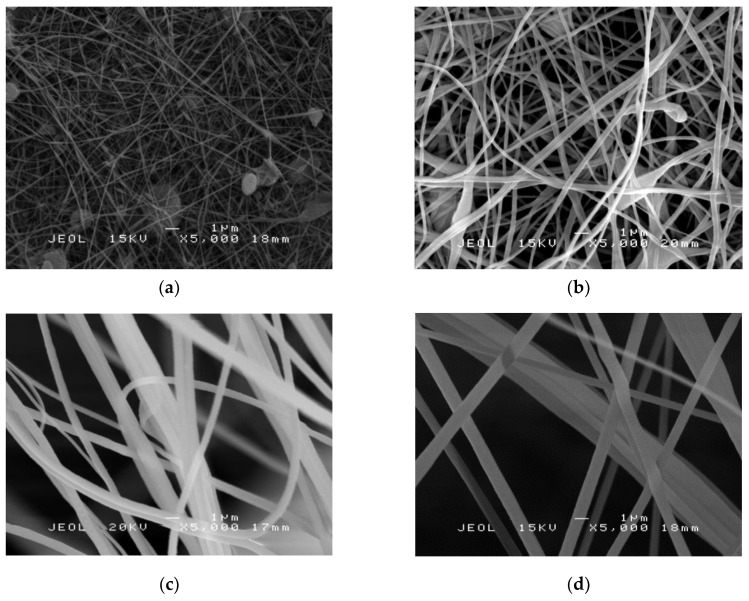
SEM micrographs of polyacylonitrile (PAN) nanofibers: (**a**) 2.5%, (**b**) 5% (**c**) 7.5% and (**d**) 10% of PAN solution (5000× magnifications).

SEM images of PAN nanofibers electrospun using various concentrations of PAN solutions are shown in Figure 1. As the concentration of PAN increased, the average diameter of the electrospun fibers also increased. The average diameter of the nanofibers was 114 ± 4 nm, 335 ± 7 nm, 558 ± 9 nm and 868 ± 15 nm for 2.5%, 5%, 7.5% and 10% PAN respectively. From the SEM micrographs, the morphology of PAN nanofibers of 2.5% consists of a significant number of beads formed despite having the smallest diameter. At a low solution concentration, a significant appearance of beads was observed, and the increase in the solution concentration transformed the beads from spherical to spindle-like fibers. A similar trend was found previously, where a decrease of total polymer concentration will lead to the formation of the beads in the electrospun fiber mats [24]. As the concentration of PAN increases to 5%, the beads occasionally appear and non-uniform fibers. A high concentration of PAN should be sufficiently high to produce smoother and uniform, thus, these nanofibers can stack more densely and the resulting fibers have a smaller pore size [25]. From the SEM images, 7.5% and 10% PAN nanofibers have smooth and uniform fibers, but 7.5% has a smaller average fiber diameter. Therefore, 7.5% was chosen for the fabrication of PAN/SL nanofibers as it shows a relatively small fiber diameter and the absence of beads. PAN 7.5% nanofibers from here onwards are denoted as PAN nanofibers only.

### 2.2. Electrospun PAN/SL Nanofibers

**Figure 2:**
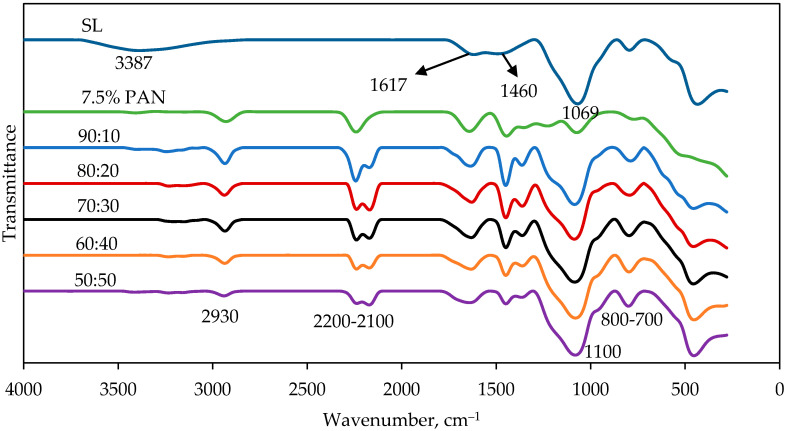
The comparison of spectra between SL, 7.5% PAN and PAN/SL nanofibers of different ratio of PAN:SL.

Electrospun PAN/SL fibers were prepared by blending PAN and sago lignin (SL) with the ratio of SL to PAN varied from 10% to 50%, using DMF as a solvent. Figure 2 shows the FTIR spectra of SL, PAN and PAN/SL nanofibers of different ratio of PAN:SL. From the IR spectra of PAN nanofibers, the vibrations of the aliphatic –CH groups (–CH, –CH_2_, and –CH_3_) are observed at around 2900 cm^−1^, 1450 cm^−1^, 1350 cm^−1^, and 1220 cm^−1^, respectively. The peak at 2200 cm^−1^ can be assigned to the nitrile group, C≡N [26]. C=O carbonyl group around 1640 cm^−1^ is due to the C=O group from the trace of DMF solvent that did not evaporate during the drying process [27]. For sago lignin, the broad peak at 3387 cm^−1^ is assigned to –OH stretching peak either alcoholic or hydroxyl group of sago lignin. The peak at 1617.76 cm^−1^ represented the C=C stretching of the benzene skeleton [28]. The peak at 1460 cm^−1^ is attributed to C–H bending vibration in methyl groups while the peak at 1069 cm^−1^ is assigned to C–O deformation in secondary and primary alcohols or aliphatic ether [29]. PAN/SL nanofibers consist of peaks from both sago lignin and PAN. The aliphatic –CH groups of PAN appeared 2930 cm^−1^ and the absorption peaks between 2200 and 2100 cm^−1^ were assigned to the C≡N nitrile group. At around 1100 cm^−1^, a peak appeared which can be assigned to C–O deformation of the benzene ring in lignin structure while at peak between 780 and 800 cm^−1^, the peaks were contributed by the bending of C–H groups of lignin’s benzene ring.

**Figure 3:**
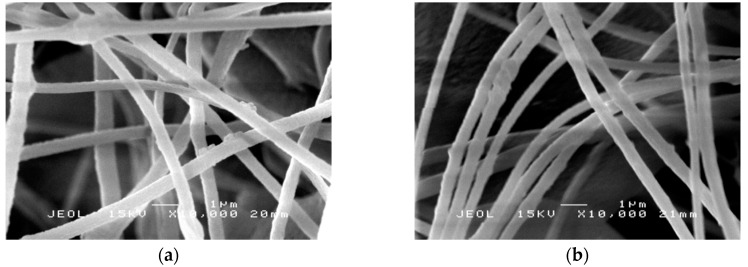

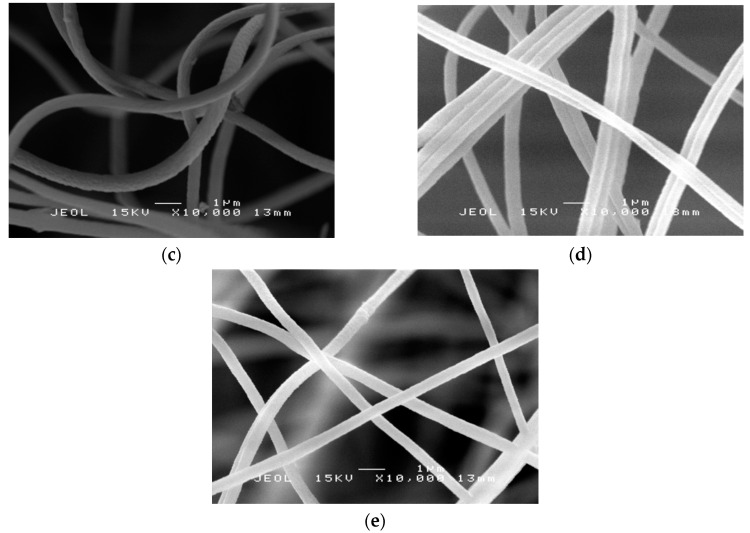
SEM micrographs of PAN/SL nanofibers: (**a**) 50:50, (**b**) 60:40 (**c**) 70:30 (**d**) 80:20 and (**e**) 90:10.

Due to the lack of chain structures and/or molecular entanglements of lignin, lignin alone could not be electrospun into nanofibers [30,31]. Therefore, lignin was blended with high molecular weight polymers, PAN in order for lignin to be electrospun. The ratio of SL was varied from 10wt% to 50wt%. Figure 3 shows SEM micrographs of PAN/SL nanofibers electrospun at different ratio of PAN/SL; 50:50, 60:40, 70:30, 80:20 and 90:10. The average diameter of PAN/SL of ratio 50:50 was 154 ± 24 nm, 60:40 was 366 ± 13 nm, 70:30 was 374 ± 38 nm, 80:20 was 402 ± 16 nm and 90:10 was 451 ± 21 nm. From the SEM micrographs, the morphology of electrospun PAN/SL nanofibers of 50:50 ratio has beads forming and breakage of fibers. For the 60:40 ratio of PAN/SL, the surface of the fibers appeared to be uneven with the occasional appearance of beads, while 70:30, 80:20 and 90:10 ratios had smooth and uniform fiber morphology. However, PAN/SL nanofibers of the 70:30 ratio of PAN/SL blend solution were chosen as the optimum concentration because its average diameter was smaller compared to 80:20 and 90:10 ratio. The bead-free nanofibers also can be tightly-packed during the electrospinning process compared to 50:50 and 60:40, and this would make the fibers possess a smaller pore size [25]. From here onwards, PAN/SL of 70:30 nanofibers will be denoted as PAN/SL nanofibers only.

### 2.3. Spectroscopic Study of The Nanofibers After Thermal Treatment (Stabilization and Carbonization Process)

**Figure 4:**
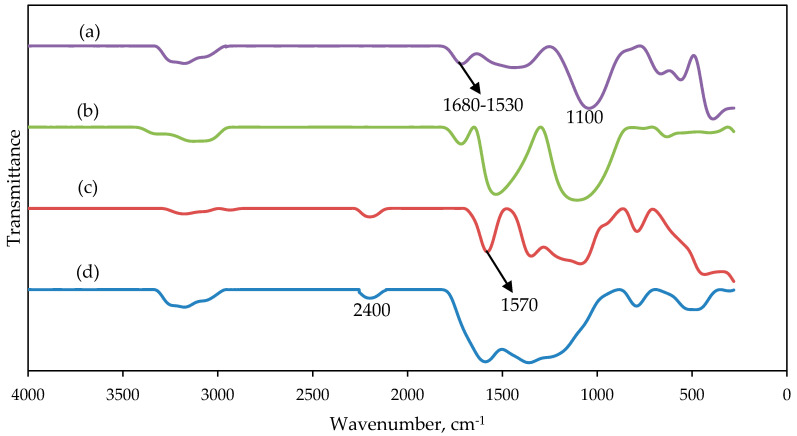
FTIR spectra of carbonized (**a**) PAN and (**b**) PAN/SL nanofibers; and stabilized (**c**) PAN and (**d**) PAN/SL nanofibers.

Figure 4 shows the FT-IR spectra of PAN and PAN/SL nanofibers after thermal stabilization and carbonization. For PAN and PAN/SL nanofibers after stabilization, the peak of C≡N nitrile group of PAN shifted to a higher wavenumber which was from between 2200–2100 cm^−1^ to 2400 cm^−1^, indicating a conversion of C≡N into C=N as a result of cyclization and cross-linking of PAN into the ladder-like structure during stabilization. The absorption peak around 1570 cm^−1^ belonged to a mix of C=N, C=C, and N–H groups and the appearance of C=C was due to dehydrogenation [32]. After carbonization, the C≡N nitrile group disappeared because at a higher temperature (carbonization process) the functional groups including NH_3_, HCN, and N_2_ disappeared. The peak between 1680 cm^−1^ and 1530 cm^−1^ was assigned to the stretching and bending of C=C groups while the absorption peak around 1100 cm^−1^ was due to C–H deformations [33].

### 2.4. Morphology Study of PAN Nanofibers and PAN/SL Nanofibers After Stabilization and Carbonization Process

**Figure 5:**
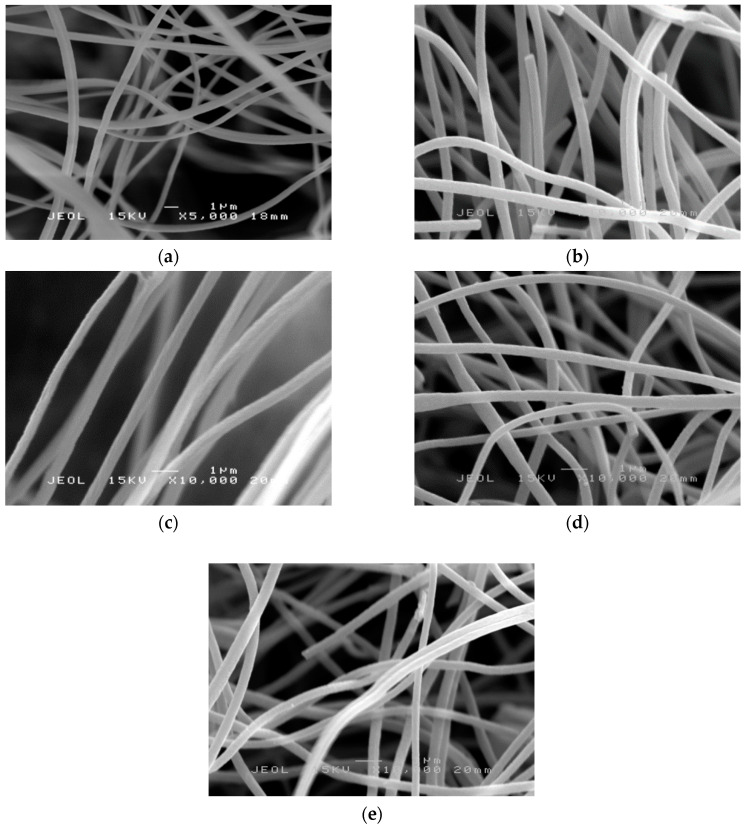
SEM micrographs of PAN nanofibers: (**a**) after stabilization and (**b**) after carbonization (5000× magnification), PAN/SL nanofibers: (**c**) after stabilization and (**d**) after carbonization (10,000× magnification) and (**e**) PAN/SL activated CNF.

PAN nanofibers and PAN/SL nanofibers underwent stabilization and carbonization and will be denoted as PAN CNFs and PAN/SL CNFs. In Figure 5a,b, the appearance of the nanofibers remained intact and no melting of fibers was observed after stabilization. The stabilization process prior to carbonization is an important process that influence the morphologies and the mechanical properties of the final carbon nanofibers [34]. For PAN nanofibers, after stabilization (Figure 5a), the average diameter of the fibers was 377 ± 6 nm, and after further thermal treatment at a higher temperature (carbonization (Figure 5b), the average diameter significantly decreased to 368 ± 8 nm. Overall, the average diameter of electrospun PAN fibers reduced almost 1.5 times after carbonization. The average diameter reduced significantly after these two processes (stabilization and carbonization) and might be due to the liberation of gases such as CO_2_, CO, NH_3_ and HCN and the densification of carbon atoms in the polymer chains during thermal treatment [35,36]. 

Figure 5c,d illustrates the SEM micrographs for PAN/SL nanofibers after stabilization and carbonization, respectively. The average diameter of the fibers for PAN/SL nanofibers was reduced to 371 ± 5 nm after stabilization and further reduced to 326 ± 5 nm after carbonization. In comparison to PAN CNFs, the diameter of average fibers for PAN/SL CNFs was smaller compared to PAN CNFs. This is because when PAN blended with sago lignin, the viscosity of the polymer solution decreased due to the smaller molecular weight of lignin and accompanied by the removal of hydrogen and oxygen atoms in SL structure during the carbonization [30] may contribute to the significant reduction of the diameter of the fibers of PAN/SL CNFs compared to PAN CNFs. Figure 5e represents the SEM image of PAN/SL activated CNF (ACNF). The average fiber diameter of ACNF is 323 nm. The SEM images reveal that after the carbonization and activation processes, the basic nanofibrous structure of PAN/SL remained intact but the fiber diameter shrank significantly.

### 2.5. Carbon Yield

The determination of carbon yield was conducted using TGA under an inert atmosphere according to previously reported by Thunga et al. [37]. The carbon yield of PAN CNFs and PAN/SL CNFs were obtained from the residual wt% at 1000 ℃ in the TGA thermogram. The carbon yield for PAN CNFs and PAN/SL CNFs were 55.3% and 66.51%, respectively. After blending with sago lignin, the carbon yield was increased by 11.21% which indicates that the aromatic structure of lignin contributed to the increase in carbon yield. Thunga et al. [37] also reported a similar result in which the carbon yield increased after PLA was blended with butyration lignin. At temperatures above 500 ℃, the amorphous carbon was formed and most of the benzene rings of lignin were transformed into aromatic structures and increased the densification of carbon atoms which lead to the increase in carbon yield [35,38]. PAN/SL and PAN (nanofibers, after stabilization and after carbonization) were further characterized using XRD. The results and discussion of XRD characterization are included in the Supplementary Materials.

### 2.6. Spectroscopic Study of PAN/SL ACNFs

**Figure 6:**
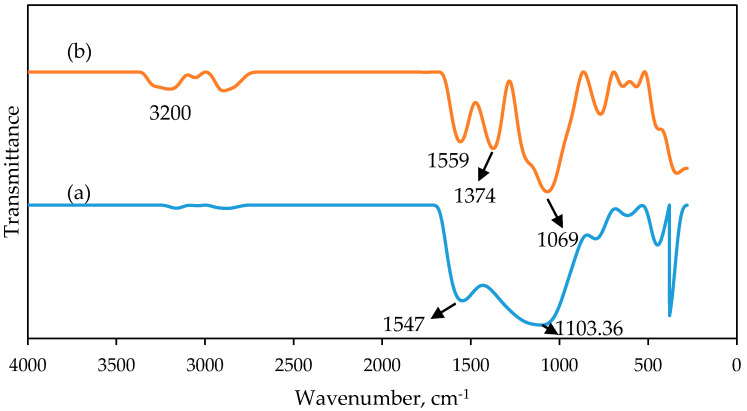
FTIR spectra of (**a**) PAN/SL CNFs and (**b**) PAN/SL ACNFs.

After the carbonization process, PAN/SL CNFs were subjected to an activation step to produce activated PAN/SL CNFs (PAN/SL ACNFs). This step modified the surface of PAN/SL CNFs using concentrated nitric acid. Figure 6 shows the IR spectra of (a) PAN/SL CNFs and (b) PAN/SL ACNFs respectively. A hydroxyl band appeared at 3200 cm^−1^ for PAN/SL ACNFs and was attributed to the presence of more –OH groups probably due to phenolic groups after modification with HNO_3_ [39]. Peaks at 1559 cm^−1^ and 1547 cm^−1^ were observed for PAN/SL ACNFs and CNFs can be assigned to highly conjugated carbonyls (quinone groups) and C=C stretching vibrations of aromatic rings [40,41]. This peak demonstrated that the C=C carbon backbone of CNFs was not affected even after modification with nitric acid. A new peak was observed for the PAN/SL ACNFs at 1374 cm^−1^ belong to symmetric NO_2_ stretch vibrations which indicates that NO_2_ groups were successfully introduced on the surface of carbon fibers upon HNO_3_ treatment [42]. The peaks at 1069 cm^−1^ and 1103 cm^−1^ were assigned to C–O deformations which can be observed for both PAN/SL ACNFs and CNFs.

### 2.7. BET Analysis of PAN/SL CNFs and PAN/SL ACNFs

**Figure 7:**
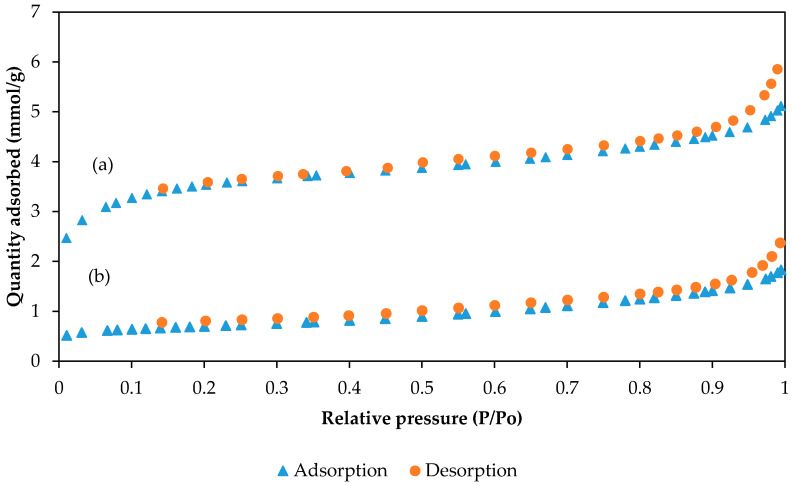
N_2_ adsorption-desorption isotherms of (**a**) PAN/SL CNFs and (**b**) PAN/SL ACNFs.

Figure 7 shows the N_2_ adsorption–desorption isotherms of (a) PAN/SL CNFs and (b) PAN/SL ACNFs. From the graph, N_2_ adsorption–desorption was lower for PAN/SL ACNFs which might be due to the limited amount of pores necessary for N_2_ adsorption. Table 1 summarizes the results of N_2_ adsorption–desorption isotherms of PAN/SL CNFs and PAN/SL ACNFs. These data demonstrate that after activation with HNO_3_, the total surface area was significantly decreased from 292.24 to 57.37 m^2^/g accompanied by a widening of the average pore diameter. The widening of average pore diameter after activation suggests the effective attack by nitric acid on the micropores, resulting in the partial destruction of their structure [43,44]. The attachment of oxygenated groups at the entrance and/or on the walls of micropores might destruct the walls of the pores and lead to the decreased in the total surface area and pore volume of the adsorbent [45].

### 2.8. XPS Analysis of PAN/SL ACNFs

**Figure 8:**
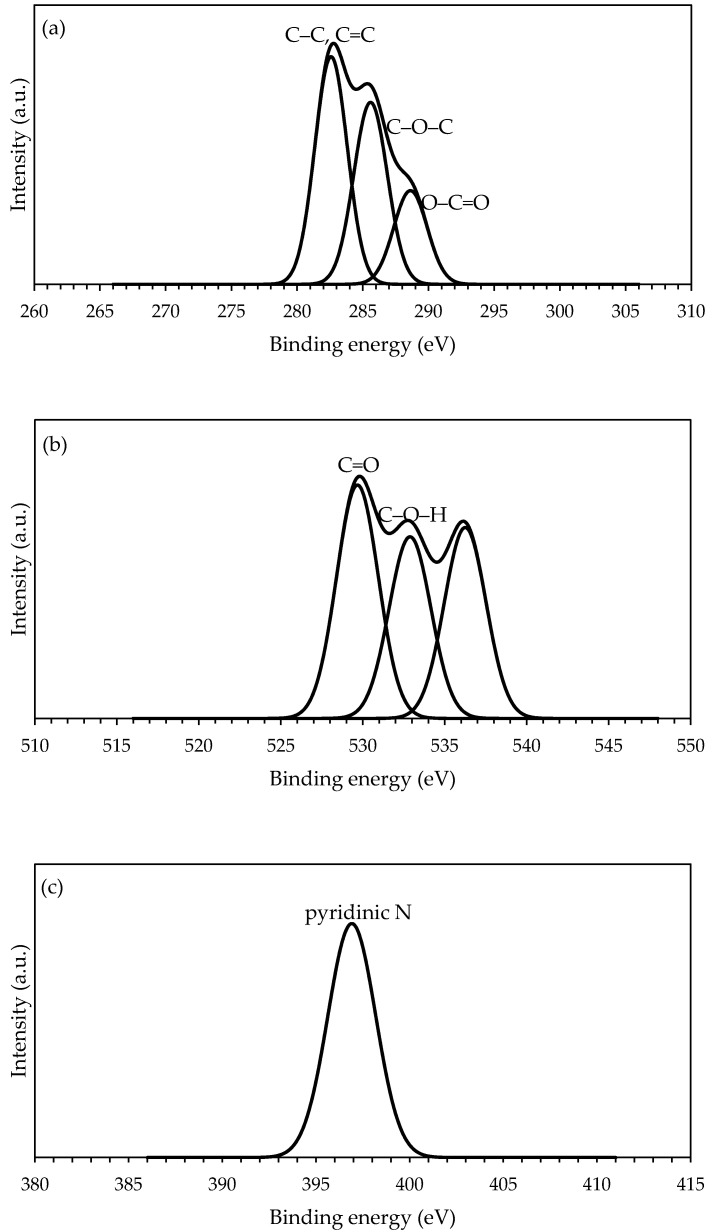
(**a**) C_1s_, (**b**) O_1s_, and (**c**) N_1s_ spectra of PAN/SL ACNFs.

The activation of PAN/SL ACNFs with nitric acid was further characterized using XPS analysis to study the chemical structure and elemental composition on its surface [26]. The C_1s_, O_1s_, and N_1s_ spectra are shown in Figure 8. The C_1s_ binding energy (BE) of PAN/SL ACNFs composed of three components assigned to carbon atoms of the polymer chain. BE of C1s that was located at 283.6 eV was assigned to C–C and C=C bonds of a graphitic structure [46]. The BE of this band was not the typical carbon in graphitic structure as this band was broadened toward lower BE which might be due to a significant contribution of the C–H bonding in the lignin [46]. The BE at peaks 285.6 and 288.8 eV are due to C–O–C (carbon in phenolic, alcohol, or ether) and O–C=O, respectively suggesting the presence of a variety of oxygenated functional groups in ACNFs [39]. The XPS O_1s_ of PAN/SL ACNFs consist of three BE bands. The band at 529.7 eV was assigned to C=O groups while the band at 532.9 eV was due to oxygen in phenolic groups, C–O–H which is common in the lignin structure [46]. BE at band 536.2 eV was caused by the adsorbed water and/or oxygen [39]. On the other hand, XPS N_1s_ only consists of one band at 397.1 eV that was assigned to pyridinic N which is due to the nitric acid treatment [47].

### 2.9. Adsorption of Pb(II) Ions

**Figure 9:**
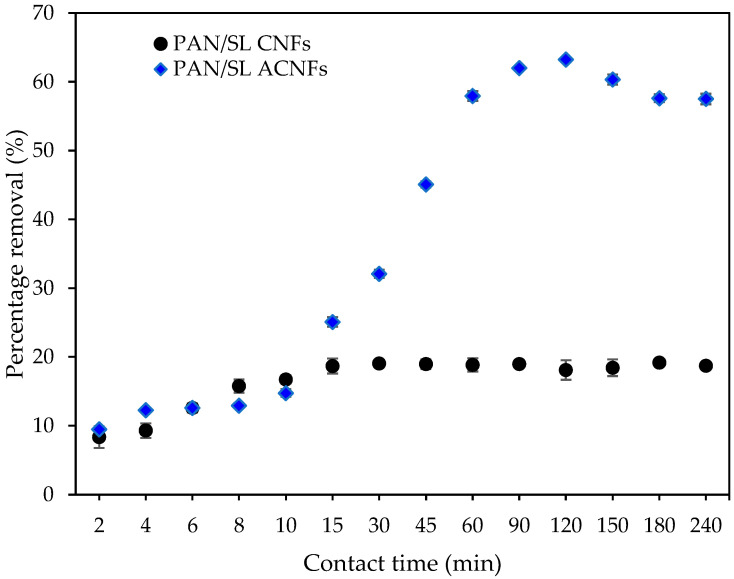
Percentage removal of Pb(II) ions for PAN/SL CNFs and PAN/SL ACNFs. Conditions: [Pb(II)] = 100 mg/L, dosage of adsorbent = 25 mg, pH = 5.

Figure 9 shows the percentage removal of Pb(II) ions for PAN/SL CNFs and PAN/SL ACNFs. Although the PAN/SL CNF has a higher surface area, the adsorption of Pb(II) ions on PAN/SL ACNF (63%) is three times higher than that of PAN/SL CNF (19%). The high percentage removal of Pb(II) ions of PAN/SL ACNF is due to the presence of the oxygenated surface group generated after the activation of the PAN/SL with HNO_3_, as evidenced by FTIR and XPS analyses. The attachment of surface oxygen groups decreases the hydrophobicity of the adsorbent surface, thus improves the interaction with Pb(II) ions and promotes the Pb(II) ion adsorption on the PAN/SL ACNF [44]. 

#### 2.9.1. Effect of Contact Time

**Figure 10:**
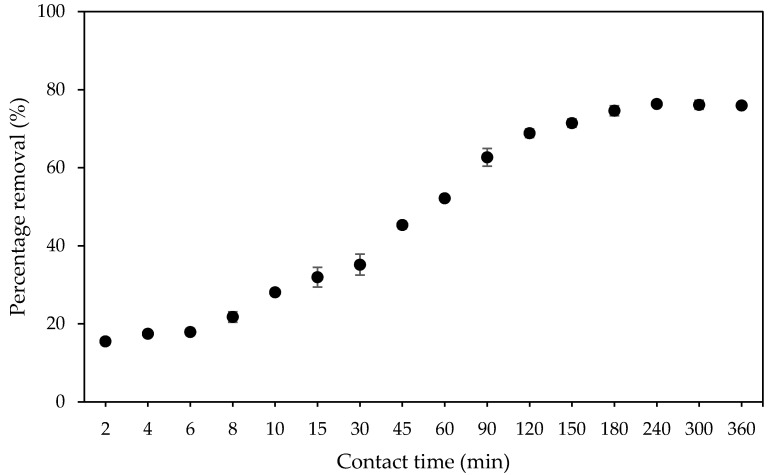
Effect of contact time on the percentage removal of Pb(II) ions. Condition: [Pb(II)] = 100 mg/L, dosage of adsorbent = 40 mg, pH = 5.

Since the time taken for the adsorption process to achieve equilibrium is of considerable significance to develop economical heavy metal adsorbents, the effect of contact time on the percentage removal of Pb(II) ions by PAN/SL ACNFs was investigated. As depicted in Figure 10, the percentage removal of Pb(II) ions increased rapidly up to 120 min. It then gradually decreased until equilibrium at 240 min with Pb(II) ions removed at a rate of 76%. The rapid adsorption at the early stage was due to the availability of a large number of vacant adsorption sites, which lead to the increase of adsorbate accumulated on the carbon nanofibers surface. The gradual decrease in the adsorption at a later stage was due to repulsive forces between the adsorbed Pb(II) ions and the Pb(II) ions in the solution as the surface became saturated and reached equilibrium [48]. 

#### 2.9.2. Effect of Adsorbent Dosage

**Figure 11:**
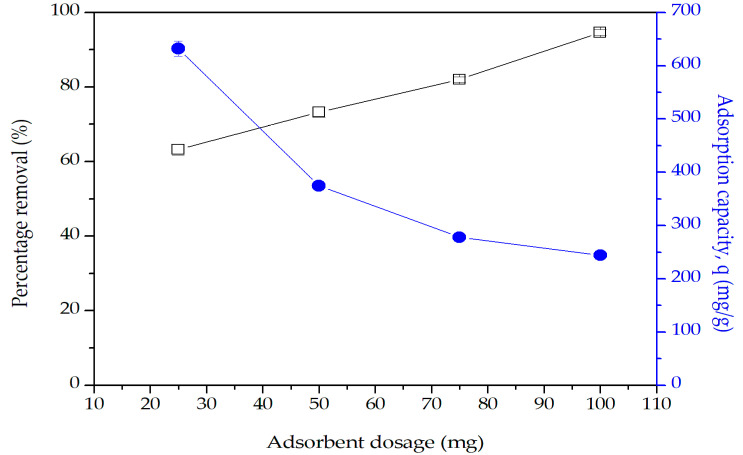
Effect of adsorbent dosage on the percentage removal and adsorption capacity, q. Condition: [Pb(II)] = 100 mg/L, pH = 5, contact time = 120 min.

The effect of adsorbent dosage on the percentage removal of Pb(II) ions and the adsorption capacity, q is illustrated in Figure 11. The percentage removal of Pb(II) ions increased from 63% to 95% with increasing adsorbent dosages. As the adsorbent dosage increases, the number of adsorption sites also increases, leading to higher percentage removal of Pb(II) ions. The adsorption capacity, q, however, decreased with increasing adsorbent dosage. The decrease is due to the increasing number of unoccupied adsorption sites as the ratio of Pb(II) ions to the adsorption sites decreases with increasing adsorbent dosage [49]. Based on both the percentage removal and the q values, the best adsorbent dosage for the adsorption study is 40 mg. 

#### 2.9.3. Effect of Initial Concentration of Pb(II) Solutions

**Figure 12:**
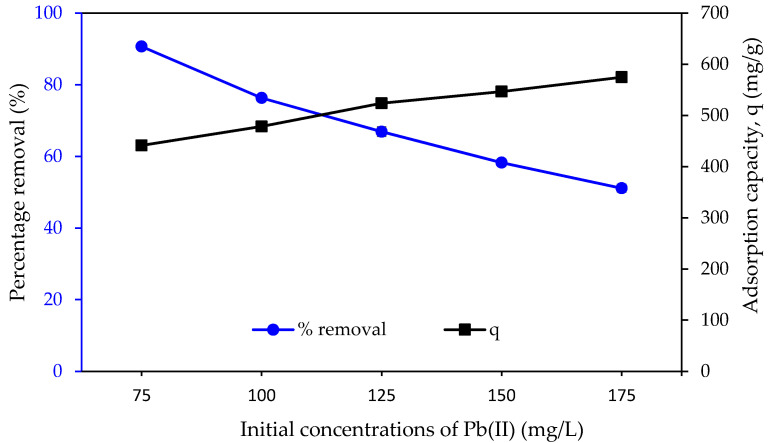
Effect of initial Pb(II) concentration on the percentage removal. Condition: pH = 5, dosage of adsorbent = 40 mg, contact time = 240 min.

Figure 12 shows the effect of various initial Pb(II) concentrations on the percentage of removal by PAN/SL ACNFs. As the concentration of Pb(II) ion increases, the percentage removal decreases while the adsorption capacity increases. With a fixed mass of adsorbent used, the ratio of adsorption sites to Pb(II) ions changes with an increasing initial Pb(II) ion solution. At a low Pb(II) concentration, the surface area and the availability of adsorption sites were relatively high, and Pb(II) ions were easily adsorbed and removed from the solution. At a higher concentration of PB(II) solution, the total available adsorption sites were limited, thus, resulting in a decrease in percentage removal of Pb(II) ions. At a higher concentration of Pb(II) solution, a higher driving force is available to overcome the resistance of Pb(II) ion between the aqueous and solid phases, which leads to an increase in adsorption capacity. At a higher concentration of 125 mg/L, the change in adsorption capacity was relatively small; thus, 125 mg/L was chosen to be used for other optimization parameters.

#### 2.9.4. Effect of pH

**Figure 13:**
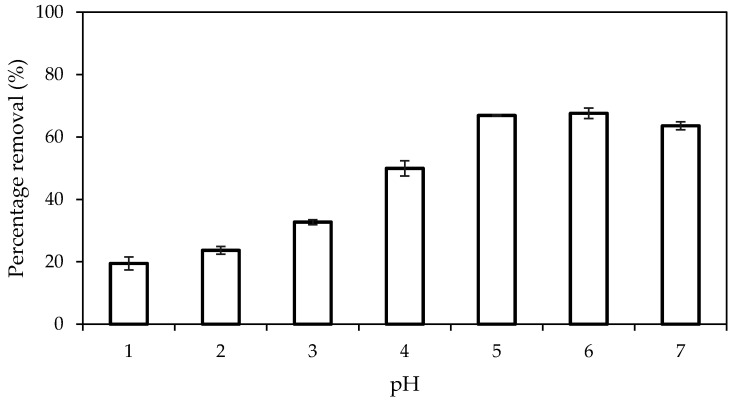
Effect of pH on the percentage removal of Pb(II) ions. Condition: dosage of adsorbent = 40 mg, contact time = 240 min, [Pb(II)] = 125 mg/L.

The adsorption of Pb(II) ions on PAN/SL ACNFs at different pH values is affected by the surface properties of the adsorbent and the speciation of the metal ions. The pHpzc of the adsorbent, as determined using the pH drift method, was 3.8, which indicates that the surface of the adsorbent was positively charged at pH below 3.8 and negatively charged at pH beyond 3.8. Pb(II) ions are known to precipitate as Pb(OH)_2_ at pH above 7. The effect of the initial pH solution, conducted at a pH range of 1 to 7, on the removal of Pb(II) ions is as shown in Figure 13. The percentage removal of Pb(II) ions was low in very acidic conditions (pH 1–3) due to electrostatic repulsion between the Pb(II) ions and the positively charged surface of the adsorbent. Besides, competition for the adsorption sites between the abundantly available H^+^ ions and Pb(II) ion can also lead to a reduction in percentage removal. The removal of Pb(II) is more significant at a pH range of 4 to 7, with the highest removal of 67% observed at pH 5–6. The enhancement of the percentage removal was due to the electrostatic attraction between the Pb(II) ion and the negatively charged surface of the adsorbent.

#### 2.9.5. Adsorption Kinetics and Isotherm

The adsorption mechanism of Pb(II) was investigated by fitting the experimental adsorption data to several kinetic models, namely, pseudo-first-order, pseudo-second-order, and intraparticle diffusion models. The linearized form of the pseudo-first-order and pseudo-second-order is as shown in Equations (1) and (2), respectively;

(1) $$\ln\left(q_{e}{-\;q}_{t}\right){=\mathrm{lnq}}_{e}{\;-\;k}_{1}t\;$$

(2) $$\frac{t}{q_{t}}=\frac{1}{k_{2}q_{e}^{2}}+\frac{1}{q_{e}}$$

where k_1_ and k_2_ are the rate constants, and q_t_ and q_e_ are the amounts of Pb(II) adsorbed at time t and equilibrium, respectively. 

Table 2 summarizes the adsorption capacities, q_e_, rate constants, and the correlation coefficient (R^2^) values at different initial concentrations of Pb(II) solutions. The R^2^ value of the pseudo-second-order model is closer to unity. Besides, the experimental q_e_ is also comparable to that of the calculated q_e_ of the pseudo-second-order model. These results show that the kinetics of Pb(II) adsorption onto PAN/SL ACNFs can be described well by the pseudo-second-order model, suggesting that the adsorption process is governed by the availability of the adsorption sites rather than the concentration of the Pb(II) ions [50].

As shown in Table 2, the second-order rate constant, k_2_, increased with increasing Pb(II) concentration up to 125 mg/L, before decreasing with further increases in the initial Pb(II) concentration. The initial increase of k_2_ may be due to increase driving force for mass transfer with increasing Pb(II) concentrations. However, at higher Pb(II) concentration, although the q_e_ increased, the k_2_ decreased. This decrease could likely be due to competitive adsorption between Pb(II) ions (highly concentrated) towards the limited adsorption sites and diffusion of metal ions from the surface to the interior adsorption sites of the adsorbent.

**Figure 14:**
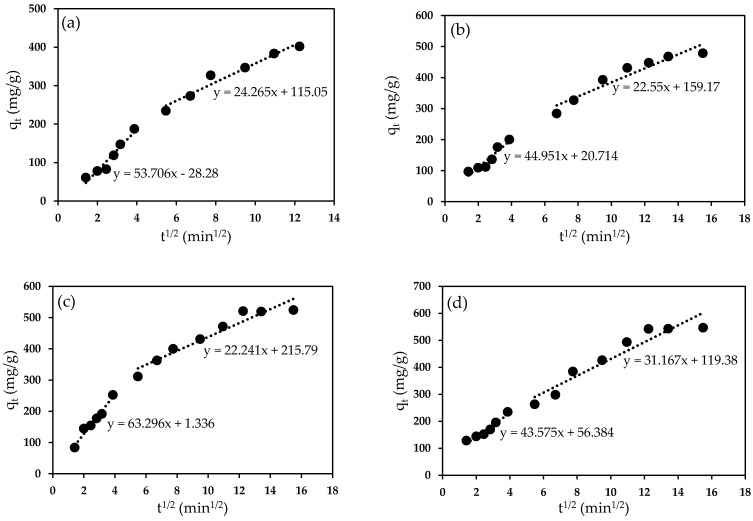

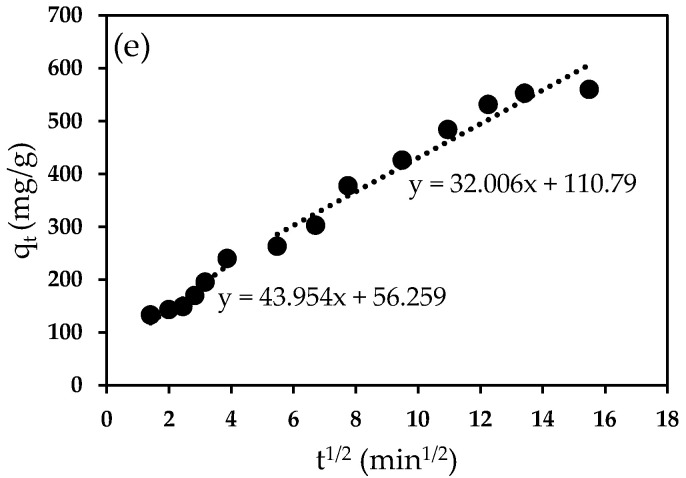
Intra-particle diffusion plot for adsorption of Pb(II) ions onto PAN/SL ACNFs at different initial Pb(II) concentrations; (**a**) 75, (**b**) 100, (**c**) 125, (**d**) 150 and (**e**) 175 mg/L. Condition: contact time = 240 min, dosage of adsorbent = 40 mg, pH = 5.

The contribution of diffusion in the adsorption mechanism was analyzed using the intra-particle diffusion model. The linearized form of the intra-particle diffusion model is expressed in Equation (3) as follows:

(3) $$q_{t}\;=\;k_{\mathrm{id}}t^{\text{½}}\;+\;C$$

where k_id_ is the intra-particle diffusion rate constant (mg/g.min^1/2^) and C is the intercept which is related to the boundary layer thickness. Figure 14 illustrates the intra-particle diffusion multilinear plot for adsorption of Pb(II) ions onto PAN/SL ACNFs at different initial Pb(II) concentrations, suggesting a multistep adsorption process. The first step is due to the adsorption of Pb(II) on the external surface of the adsorbent via boundary layer diffusion [51]. The second step is the gradual adsorption stage, which can be related to the diffusion of the Pb(II) ions into the pores of the adsorbent, where intra-particle diffusion is the rate-controlling step [52]. The intra-particle diffusion rate, k_id_, (Table 3) at Pb(II) concentrations above 125 ppm is much higher than that below 125 ppm. This observation corresponds to the greater driving force for mass transfer at higher Pb(II) concentration, which in turn leads to an increase in the q_e_ value.

The adsorption isotherm is essential for validating an accurate prediction of adsorption parameters and quantitative analysis of adsorption behavior for various adsorbent systems and conditions [53]. In this study, Langmuir and Freundlich isotherm models were applied to investigate the interaction between adsorbate and adsorbent. The linearized form of the Langmuir and Freundlich models is as shown in Equations (4) and (5), respectively:

(4) $$\frac{C_{e}}{q_{e}}=\frac{1}{K_{L}q_{m}}+\frac{1}{q_{m}}C_{e}$$

(5) $${\mathrm{logq}}_{e}{=\mathrm{logK}}_{F}+\frac{1}{n}{\;\mathrm{logC}}_{e}$$

where, C_e_ (mg/L) and q_e_ (mg/g) is the concentration of metal ions, and the amount of metal ion adsorbed at equilibrium, respectively, while q_m_ (mg/g) is the maximum adsorption capacity. K_L_ (L/mg) is the Langmuir constant that is related to the affinity of the binding site, whereas n and K_F_ are Freundlich constants related to adsorption intensity and adsorption capacity, respectively. As shown in Table 4, the R^2^ value for both the Langmuir and Freundlich isotherm model is near unity, indicating that the adsorption of Pb(II) ions on PAN/SL ACNFs fitted both adsorption models. However, the calculated q_e_ values of pseudo-second-order kinetics for Pb(II) concentrations of 150 and 175 ppm (Table 2) are the same as the calculated q_m_ value of Langmuir isotherm. This similarity indicates that monolayer adsorption of Pb(II) ions took place on the surface of the adsorbent. Hence, the adsorption process can be said to follow the Langmuir isotherm model with q_m_ of 588.24 mg/g and K_L_ of 0.2537 L/mg.

The adsorption of Pb(II) by PAN/SL ACNF was compared to that of other carbon-based adsorbents reported in the literature, and the results are shown in Table 5. The maximum adsorption capacity of PAN/SL ACNFs demonstrated that it had higher efficiency for the adsorption of Pb(II) from aqueous solution compared to other carbon-based adsorbents stated in the table.

## 3. Materials and Methods

### 3.1. Preparation of Electrospun PAN and PAN/SL Nanofibers

The isolation of sago lignin from sago waste was conducted in accordance with Schwanninger and Hinterstoisser [55] with a slight modification. PAN solutions were prepared by dissolving PAN with DMF as the solvent with various concentrations (2.5, 5, 7.5, and 10% (*wt/v*)) for the preparation of electrospun PAN fibers. The solutions were stirred at room temperature until homogenous solutions were obtained. Electrospun PAN/SL fibers were prepared by blending PAN with SL with a ratio of lignin to PAN varied from 10% to 50% using DMF as the solvent. The polymer blend was stirred at room temperature until the polymers were dissolved completely.

For the electrospinning process, the polymer solution was loaded into a 5 mL syringe attached with a 0.8 mm inner diameter needle. The distance between the needle tip and the aluminum foil collector was fixed at 10 cm. The electrospinning was conducted using 18 kV voltage with a 2 mL/h flow rate. The nanofibers formed on the collector was collected and dried in the oven overnight. 

### 3.2. Preparation of Carbon Nanofibers

PAN nanofiber and PAN/SL nanofibers undergone two steps of thermal treatment to transform into CNFs. The electrospun nanofibers were thermally stabilized at 250 °C for 1 h in the air. The sample was then carbonized in a tube furnace at 1000 °C for 1 h under nitrogen gas flow. The carbon nanofiber produced from PAN nanofiber and PAN/SL nanofiber was denoted as PAN CNF and PAN/SL CNF, respectively. The PAN/SL CNF was then activated, to produce PAN/SL ACNF, according to Ihsanullah et al. [56], with some minor modifications. The activation was done to decrease the hydrophobicity of the carbon adsorbent. Three hundred milliliter of 69% nitric acid was added to 2 g of PAN/SL CNF, and the mixture was heated to 80 ℃ and refluxed at 120 ℃ for 48 h under a fume hood. Once it had cooled to room temperature, about 200 mL of deionized water was added into the mixture. The PAN/SL ACNFs was recovered via filtration and washed with deionized water until the pH of the filtrate was neutral. The activated adsorbent was dried in the oven at 100 ℃ overnight. All the prepared samples were sent for characterization.

### 3.3. Characterizations of PAN/SL ACNFs

The morphology of the nanofibers was imaged using scanning electron microscopy, SEM (JEOL JSM 6400, Tokyo, Japan), and the average fiber diameter was determined using ImageJ software (downloaded at https://imagej.net/Downloads) with 200 readings per sample. The infrared spectrum of the samples, before and after activation, was recorded in the range of 4000 to 280 cm^−1^ on an attenuated total reflectance Fourier transform infrared (ATR-FTIR) spectrometer (Perkin Elmer Spectrum RXI, Waltham, MA, USA) to determine the functional groups of the samples. Thermal gravimetric analysis (TGA), Mettler Toledo Thermogravimetric Model TGA/SDTA, was used to determine the thermal stability and carbon yield of the CNFs. The samples were heated from 35 °C to 1000 °C with a heating rate of 10 °C/min under a nitrogen atmosphere, and the amount of weight loss as a function of temperature was examined. X-ray diffraction (XRD) is an ideal method for characterization and identification of crystalline phase of the nanofibers and CNFs. The powder was put in specific mould and diffraction was generated by CuKα radiation. The analysis was carried out using Shimadzu Model XRD-6000 (Tokyo, Japan) and the 2*θ* range from 2^0^ to 60^0^ with scanning rate of 2^0^/min were used. The surface area and porosity of the prepared carbon fiber were determined by N_2_ adsorption measurements at 77 K on a BELsorp Mini II. The surface area of the carbon fibers was obtained using the Brunauer-Emmet-Teller (BET) method. At the same time, the porosity (pore volume and average pore diameter) was evaluated using the Barret, Joyner, and Halenda (BJH) method. X-ray photoelectron spectroscopy (XPS) was carried out on a PHI 5000 ESCALAB MKII instrument (Chanhassen, MN, USA) with an Al Kα anode. XPS was used to examine the chemical composition of the ACNFs and the attachment of oxygenated functional groups. A monochromatic X-ray beam source at 1638.4 eV (aluminum anode) was used to scan the sample surface. All binding energies were referenced to the neutral C1s peaks at 284.8 eV. The narrow scan data were used to determine probable compounds for the C, O, and N peaks [42]. The surface charge of the adsorbent was evaluated by determining the pH point of zero charge (pHpzc). At this pH, the electrical charge density of the adsorbent surface was zero [57,58]. The pHpzc was determined using the pH drift method, with sodium chloride served as an inert electrolyte [59]. Sodium chloride (0.1 M) was placed in a series of conical flasks, and the pH of the solution was adjusted to 1–7 using 0.1 M HCl or 0.1 M NaOH. ACNFs (40 mg) were added into each of the flasks and agitated for 24 h at room temperature. After 24 h, the equilibrated solutions were decanted, and the final stabilized pH was measured using pH meter. The final pH was plotted against the initial pH and the pH at which the curve crosses the pH_initial_ = pH_final_ line was taken as pHpzc [60].

### 3.4. Batch Adsorption of Pb(II) Ions

The adsorption of Pb(II) ions by PAN CNFs and PAN/SL CNFs was studied using the batch adsorption method. The stock solution of Pb(II) ions (1000 ppm) was prepared by dissolving 1.5980 g of Pb(NO_3_)_2_ salt with deionized water in 1000 mL volumetric flask. Different initial concentrations of Pb(II) ion solutions were prepared by diluting the stock solution. The adsorption of Pb(II) was conducted by contacting 100 mg/L Pb(II) solution with 25 mg each of PAN CNF and PAN/SL CNFs at pH 5. The experiments were conducted in a 250 mL conical flask and the solution mixture was magnetically stirred at 200 rpm for 240 min at room temperature. After specified time intervals, the solution mixture was collected and analyzed using an inductively coupled plasma-optical emission spectrometer (ICP-OES, Perkin Elmer Optima 2100 DV, Shelton, CT, USA).

To optimize the adsorption process, the one factor at a time method was used. The four parameters used in the optimization experiments were adsorbent dosage (25–100 mg), contact time, initial concentration of Pb(II) solution (75–175 mg/L) and pH of the solution (1–7). The pH of the solution was adjusted to the desired value by the addition of either 1.0 M HCl or 1.0 M NaOH solution. The percentage of removal of Pb(II) and the adsorption capacity was calculated using Equations (6) and (7), respectively.

(6) $$\mathrm{Removal}\;\left(\%\right)=\frac{C_{0}\;\;\;{-\;C}_{t}}{C_{0}}\;\times \;100\;$$

(7) $$\mathrm{Adsorption}\;\mathrm{capacity},\;q=\frac{C_{0}{\;-\;C}_{t}}{m}\;\times \;V$$

where q (mg/g) is the amount of Pb(II) adsorbed on the adsorbent C_0_ (mg/L) is the initial concentration of Pb(II) ions, C_t_ (mg/L) is the concentration of Pb(II) in solution at time t, V (L) is the volume of Pb(II) ions solution, and m (g) is the mass of adsorbent.

## 4. Conclusions

In this study, PAN/SL ACNFs were successfully prepared by incorporating sago lignin into PAN via a simple and versatile technique which is electrospinning. PAN/SL ACNFs have shown significantly higher percentage removal of Pb(II) by three times after activation with HNO_3_ due to the presence of surface oxygen groups. The best-suited adsorption conditions that gave the highest percentage removal of 67%, with an adsorption capacity of 524 mg/g, were 40 mg of adsorbent dosage, 125 ppm of Pb(II) solution, pH 5, and a contact time of 240 min. It was determined that the pseudo-second-order kinetic model was well-fitted with the experimental data which indicates that the adsorption process was controlled by chemisorption. From the intra-particle diffusion multilinear plot, it was suggested that two steps of adsorption were involved in the adsorption of Pb(II) ions. Besides, the adsorption process can be said to follow the Langmuir isotherm model with q_m_ of 588.24 mg/g. These results imply that PAN/SL ACNFs are effective adsorbent for the removal of Pb(II) from aqueous solution and have the potential to be used as the adsorbent for wastewater treatment.

## Figures and Tables

**Table 1:** Textural characterization of PAN/SL CNFs and PAN/SL ACNFs.

Nanofibers	Surface Area (m^2^/g)		Pore Volume (cm^3^/g)		Average Pore Diameter (nm)
	^a^ Total	^b^ Micropore	^c^ Total	^b^ Micropore	
PAN/SL CNFs	292.24	168.27	0.163	0.0683	3.18
PAN/SL ACNFs	57.37	28.25	0.0534	0.0116	4.51

^a^ BET surface area determined by the BET method within relative pressure of P/P0 = 0.1–0.01. ^b^ Micropores surface area and pore volume were calculated by the t-plot method. ^c^ Total pore volume measured at a relative pressure of P/P0 = 0.9.

**Table 2:** Kinetics study of PAN/SL ACNFs at different initial Pb(II) concentrations. Condition: contact time = 240 min, dosage of adsorbent = 40 mg, pH = 5.

C_0_ (mg/L)	q_e_, expt. (mg/g)	Pseudo 1st Order			Pseudo 2nd Order		
		k_1_ (min^−1^)	q_e,_ calc. (mg/g)	R^2^	k_2_ (g/mg/min)	q_e_, calc. (mg/g)	R^2^
75	441.34	0.0258	427.3	0.8747	8.51 × 10^−5^	476.19	0.9986
100	478.44	0.0178	375.67	0.9257	8.75 × 10^−5^	500.00	0.9951
125	523.91	0.0256	475.61	0.9513	9.73 × 10^−5^	555.56	0.9952
150	546.66	0.0260	554.13	0.9502	7.30 × 10^−5^	588.24	0.9811
175	574.75	0.0197	506.44	0.9425	7.10 × 10^−5^	588.24	0.98

**Table 3:** Intra-particle diffusion constant for adsorption of Pb(II) ions onto PAN/SL ACNFs at different initial Pb(II) concentrations. Condition: contact time = 240 min, dosage of adsorbent = 40 mg, and pH = 5.

C_i_ (mg/L)	1st Step		2nd	
	K_id_ (mg/g.min^1/2^)	R^2^	k_id_ (mg/g.min^1/2^)	R^2^
75	53.706	0.9391	24.265	0.9573
100	44.951	0.9002	22.55	0.9124
125	63.296	0.9697	22.241	0.9244
150	43.575	0.9443	31.167	0.9089
175	43.954	0.9091	32.006	0.9432

**Table 4:** Adsorption isotherms study of PAN/SL ACNFs. Condition: contact time = 240 min, dosage of adsorbent = 40 mg, initial [Pb(II)] = 125 mg/L, pH = 5.

**PAN/SL ACNFs**	**Langmuir Isotherm**			**Freundlich Isotherm**		
	**q_m_ (mg/g)**	**K_L_ (L/mg)**	**R^2^**	**K_F_ (mg/g)**	**1/n**	**R^2^**
	588.24	0.2537	0.999	339.94	0.1132	0.9902

**Table 5:** Comparison of maximum adsorption capacity of PAN/SL ACNF with other literature results.

Adsorbents	q_m_ (mg/g)	References
PAN/SL ACNFs	588.24	Present study
PAN/ZnO ACNF	120.3	[54]
Lignin-grafted CNT	258	[16]
Palm oil mill effluent AC	94.34	[15]

## Supplementary Materials

The following are available online at https://www.mdpi.com/1420-3049/25/13/3081/s1. Figure S1: XRD pattern for PAN/SL (a) carbonized, (b) stabilized and (c) nanofibers, and PAN (d) carbonized, (e) stabilized and (f) nanofibers.
