# Supplementary material for: Effective Removal of Pb(II) Ions by Electrospun PAN/Sago Lignin-Based Activated Carbon Nanofibers
Source: Molecules. 2020 Jul 6;25(13):3081. doi: 10.3390/molecules25133081 (PMC7411989; doi:10.3390/molecules25133081)
Supplement: Supplementary file 1 [file molecules-25-03081-s001.pdf]

Supplemetary Materials on

Effective Removal of Pb(II) Ions by Electrospun PAN/Sago Lignin-based Activated Carbon Nanofibers

Nurul Aida Nordin [1], Norizah Abdul Rahman [1,2*] and Abdul Halim Abdullah [1,3]

[1] Department of Chemistry, Faculty of Science, Universiti Putra Malaysia, 43400 UPM Serdang, Selangor, Malaysia.

[2] Materials Processing and Technology Laboratory, Institute of Advanced Technology, Universiti Putra Malaysia, 43400 UPM Serdang, Selangor, Malaysia.

[3] Materials Synthesis and Characterization Laboratory, Institute of Advanced Technology, UniversitiPutra Malaysia, 43400 UPM Serdang, Selangor, Malaysia.

*Correspondence: a_norizah@upm.edu.my

Supplementary Materials, 2 pages with 1 figure.

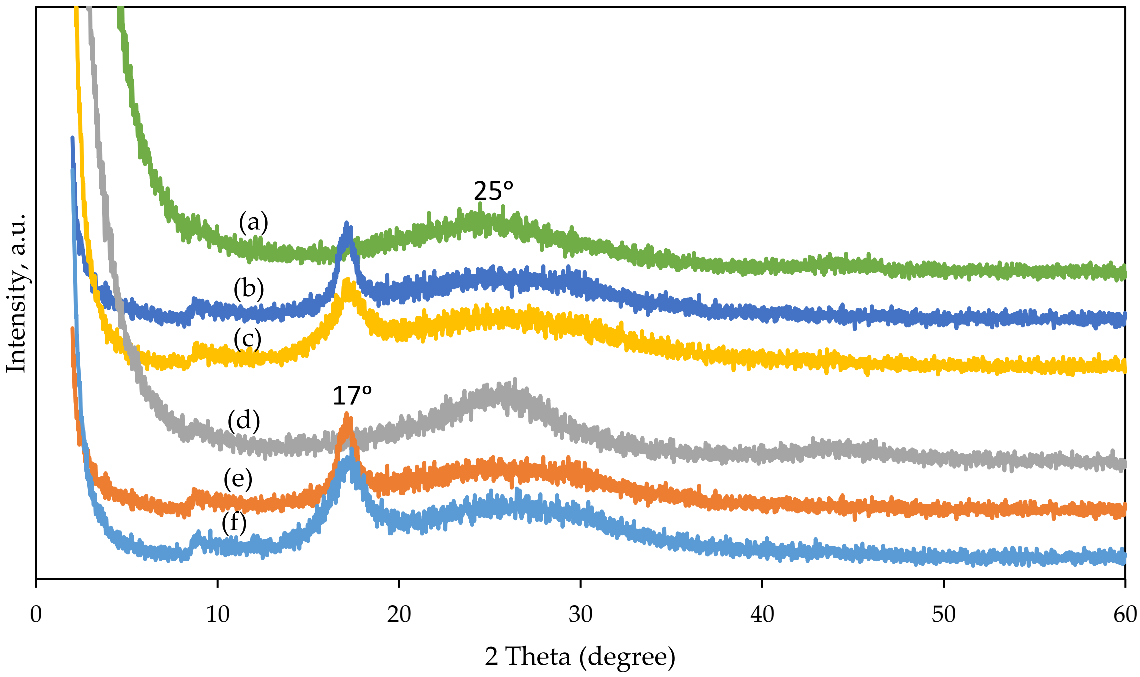

Figure S1. XRD pattern for PAN/SL (a) carbonized, (b) stabilized and (c) nanofibers, and PAN (d) carbonized, (e) stabilized and (f) nanofibers.

PAN/SL and PAN (nanofibers, after stabilization and after carbonization) were further characterized using XRD. From Figure S1, the XRD diffraction pattern of the nanofibers before and after stabilization show a strong peak at around 17° which can be ascribed to (100) crystallographic planes of PAN. However, this peak was decreased after carbonization due to the formation of ladder-like polymer structures of PAN [1]. After carbonization, a weak and broad peak was observed at around 2θ = 25° which attributed to the (002) planes [2,3] which indicates that the carbon is amorphous [4] and the nanofibers were fully converted into carbon nanofibers.

References

1. Seo, D.K.; et al. Preparation and characterization of the carbon nanofiber mat produced from electrospun PAN/Lignin precursors by electron beam irradiation. *Rev. Adv. Mater. Sci.* **2011**. *28*, 4.
2. Ji, L.; et al. Fabrication of carbon nanofiber-driven electrodes from electrospun polyacrylonitrile/polypyrrole bicomponents for high-performance rechargeable lithium-ion batteries. *Journal of Power Sources* **2010**, *195*, 2050–2056.
3. Zhu, J.; et al. Nitrogen-doped carbon nanofibers derived from polyacrylonitrile for use as anode material in sodium-ion batteries. *Carbon*, **2015**, *94*, 189–195.
4. Wang, Z.; et al. Functionalized N-doped interconnected carbon nanofibers as an anode material for sodium-ion storage with excellent performance. *Carbon*, **2013**, *55*, 328–334.
